# Response of the soil microbial community to petroleum hydrocarbon stress shows a threshold effect: research on aged realistic contaminated fields

**DOI:** 10.3389/fmicb.2023.1188229

**Published:** 2023-06-14

**Authors:** Wenjuan Jia, Lirong Cheng, Qiuyang Tan, Yueqiao Liu, Junfeng Dou, Kai Yang, Qing Yang, Senjie Wang, Jing Li, Geng Niu, Lei Zheng, Aizhong Ding

**Affiliations:** ^1^College of Water Sciences, Beijing Normal University, Beijing, China; ^2^Experiment and Practice Innovation Education Center, Beijing Normal University at Zhuhai, Zhuhai, China; ^3^Beijing Geological Environment Monitoring Institute, Beijing, China; ^4^Beijing Municipal No.4 Construction Engineering Co., Ltd., Beijing, China

**Keywords:** soil multifunctionality, microbial diversity, microbial co-occurrence network, keystone taxa, niche characteristics

## Abstract

**Introduction:**

Microbes play key roles in maintaining soil ecological functions. Petroleum hydrocarbon contamination is expected to affect microbial ecological characteristics and the ecological services they provide. In this study, the multifunctionalities of contaminated and uncontaminated soils in an aged petroleum hydrocarbon-contaminated field and their correlation with soil microbial characteristics were analyzed to explore the effect of petroleum hydrocarbons on soil microbes.

**Methods:**

Soil physicochemical parameters were determined to calculate soil multifunctionalities. In addition, 16S high-throughput sequencing technology and bioinformation analysis were used to explore microbial characteristics.

**Results:**

The results indicated that high concentrations of petroleum hydrocarbons (565–3,613 mg•kg^−1^, high contamination) reduced soil multifunctionality, while low concentrations of petroleum hydrocarbons (13–408 mg•kg^−1^, light contamination) might increase soil multifunctionality. In addition, light petroleum hydrocarbon contamination increased the richness and evenness of microbial community (*p* < 0.01), enhanced the microbial interactions and widened the niche breadth of keystone genus, while high petroleum hydrocarbon contamination reduced the richness of the microbial community (*p* < 0.05), simplified the microbial co-occurrence network, and increased the niche overlap of keystone genus.

**Conclusion:**

Our study demonstrates that light petroleum hydrocarbon contamination has a certain improvement effect on soil multifunctionalities and microbial characteristics. While high contamination shows an inhibitory effect on soil multifunctionalities and microbial characteristics, which has significance for the protection and management of petroleum hydrocarbon-contaminated soil.

## Highlights

– Low concentrations of petroleum hydrocarbons increase soil microbial diversity and microbial co-occurrence network connectivity.
– High concentrations of petroleum hydrocarbons simplify the microbial co-occurrence network.
– Petroleum hydrocarbon contamination may promote the cooperation among microbial species.
– High concentrations of petroleum hydrocarbons increase the niche overlap of keystone taxa in the microbial community.

## 1. Introduction

The soil microbial community is the most biologically diverse community in the biosphere (Sokol et al., 2022). These bacterial species communicate with each other through material, energy, and information exchanges, creating complex interactions (Fallahi et al., 2021). In addition, they play important roles in ecological processes in terrestrial ecosystems, including soil decomposition, nutrient cycling, pollutant degradation and crucial ecosystem services maintenance in the face of environmental changes (Khamehchiyan et al., 2007). However, these functions are now strongly affected by anthropogenic pressures, among which petroleum hydrocarbons are one of the most concerning. Petroleum hydrocarbons are essential basic materials for human existence and development. According to the annual report of the OPEC, global oil consumption reached 96.65 mb/d, and global oil production reached 68.73 mb/d in 2021. With the mass production and wide use of petroleum products, a large number of petroleum hydrocarbons are released into the environment during production, transportation, storage or consumption and will eventually be adsorbed onto soil pores (Gao et al., 2022), posing a serious threat to soil ecological functions (Karthick et al., 2019).

The effects of petroleum hydrocarbons on soil microbes are mainly reflected in two aspects. On the one hand, petroleum hydrocarbons can change the environment which soil microbes live. Petroleum hydrocarbons will increase the soil organic carbon content and C/N ratio (Andrade et al., 2004). In addition, petroleum hydrocarbons can affect the physiochemical properties of soil (Ossai et al., 2020). Due to the low density, strong adhesion and low emulsification capacity of petroleum hydrocarbons, they can significantly change the permeability, water holding capacity and soluble salt content of soil (Khanna et al., 2013). Some studies have shown that petroleum hydrocarbons can also change the soil pH and reduce the soil available phosphorus content (Wang et al., 2013; Li et al., 2020).

On the other hand, petroleum hydrocarbons can affect the metabolic activities of soil microbes and reshape the structure and function of the microbial community (Jiao et al., 2016; Baoune et al., 2018). Petroleum hydrocarbons are refractory organic matter and have direct or indirect toxic effects on many microbes in soil (Caravaca and Roldán, 2003). Petroleum hydrocarbons can damage the cell membrane of microbes nonspecifically, causing changes in cell membrane fluidity, integrity and function and inhibiting some metabolic functions of microbes. Only a portion of the microbes in the community can adapt to petroleum hydrocarbon-contaminated environments (Xu et al., 2022), which will grow rapidly and have high activity under the stimulation of petroleum hydrocarbons (Jia et al., 2016; Li et al., 2020).

All of these effects will change the composition, diversity and structure of soil microbes and then affect their ecological functions. However, the responses of resource consumption, growth, reproduction and interactions of different species to environmental changes differ greatly (Kim et al., 2021; Xu et al., 2022). When stressed by petroleum hydrocarbons, bacteria in soil may grow or die, increasing or decreasing in abundance. While the comprehensive response of diversity and co-occurrence of soil microbial community to petroleum hydrocarbon pollution and their contribution on soil multifunctionality are ambiguous due to the challenges of characterizing such complex communities. Therefore, we believe further insight into the structure, diversity, keystone taxa and biotic interactions of the microbial community in petroleum hydrocarbon-contaminated soil will help to improve our understanding of the mechanisms of soil microbes and their soil functions and provide information for contaminated field restoration. Observations under realistic field settings are necessary to discern the responses of soil functions and microbial community traits to petroleum hydrocarbons.

**Figure 1:**
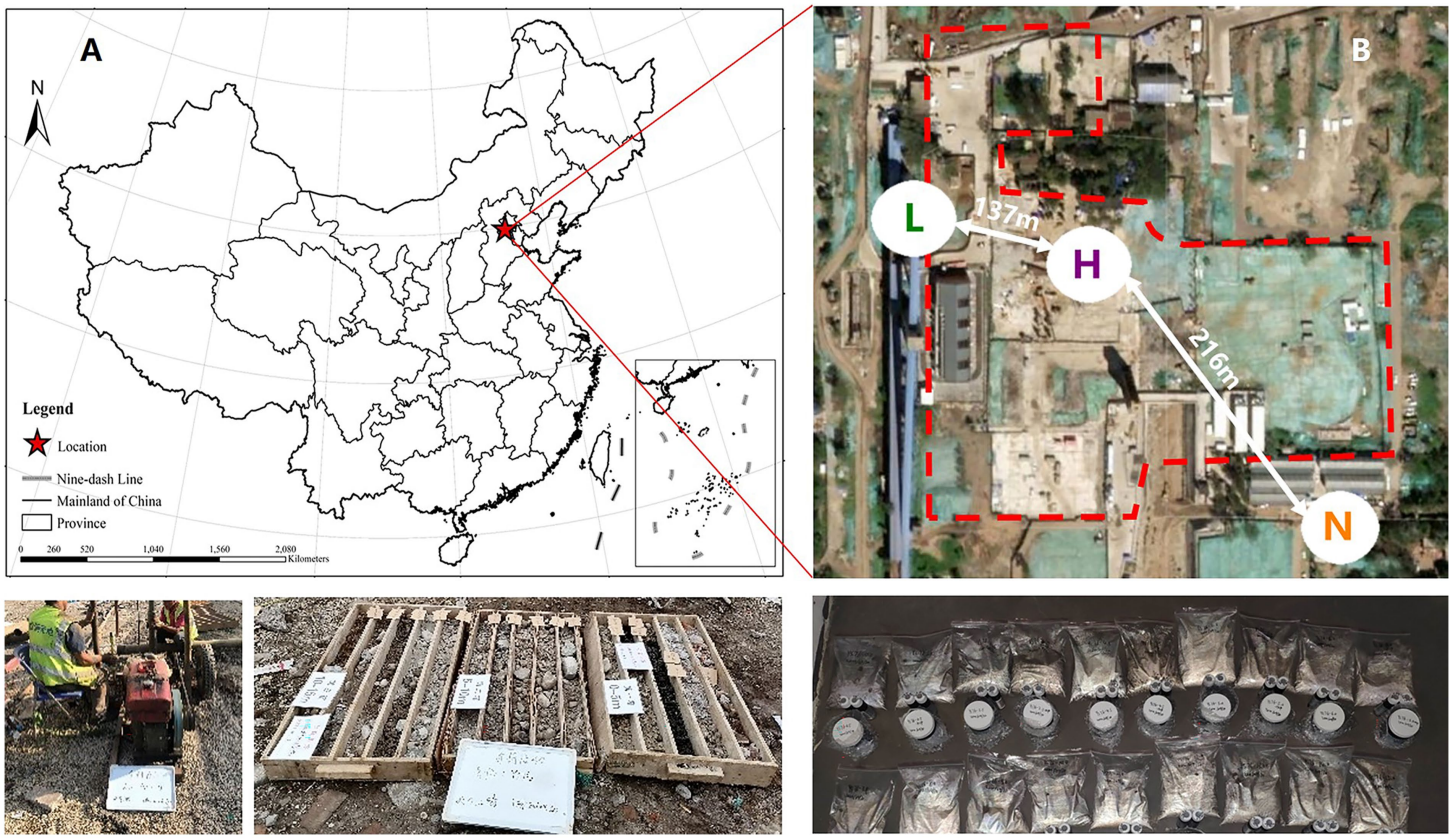
**(A)** Map of soil sampling locations in Beijing, China. **(B)** Sampling area of highly contaminated soil (H), where the concentrations of petroleum hydrocarbons were 565–3,613 mg•kg^−1^, lightly contaminated soils (L), where the concentrations of petroleum hydrocarbons were 13–408 mg•kg^−1^, and uncontaminated soils (N).

Among soil microbes, bacteria are the most important for petroleum hydrocarbons degradation and has been extensively studied by a large number of literatures (Huang et al., 2021; Ma et al., 2021; Sheng et al., 2021; Huang et al., 2022; Xu et al., 2022). Therefore, we only took bacteria as the research object in this study. Thus, with the help of 16S rRNA high-throughput sequencing and bioinformatics methods, we set the aims of this research to emphasize the variations in key species and their niches and the responses of the structure, diversity and biotic interactions of the soil microbial community to petroleum hydrocarbon stress by sampling in an aged petroleum hydrocarbon-contaminated realistic field and examining how the composition, diversity and network complexity of soil microbial communities differ between noncontaminated and varying contaminated level samples. We also determined whether microbial communities under petroleum hydrocarbon stress are associated with soil multifunctionality. We hypothesized that petroleum hydrocarbons have two effects on the microbial community: (a) as carbon sources that improve bacterial growth and (b) as toxic substances that inhibit bacterial growth. To test this hypothesis, we sampled from 3 different sites. Site A was not contaminated as a control, Site B was low-level contaminated and Site C was high-level contaminated. We examined whether the responses would be similar in such different contexts. The study was conducted in Beijing, China. These 3 sites are separated by a distance of several hundred meters (Figure 1).

At each site, we measured a range of soil physical, chemical and microbial properties and soil functional parameters. We also quantified the composition and diversity of soil bacteria by using 16S rRNA gene amplicon sequencing. These data were used to construct co-occurrence networks to explore potential biotic interactions and identify microbial keystone taxa. According to further correlation analysis of these parameters, we demonstrated that (1) high petroleum hydrocarbon contamination reduced soil multifunctionality, while light petroleum hydrocarbon contamination increased soil multifunctionality. (2) High petroleum hydrocarbon contamination reduced microbial community diversity, simplifying microbial networks and widening the niche overlap of keystone taxa, while light petroleum hydrocarbon contamination increased microbial community diversity, enhancing microbial networks. (3) The multifunctionality of soils polluted by petroleum hydrocarbon was significant correlation with diversity and co-occurrence of soil microbial community.

## 2. Materials and methods

### 2.1. Site description and soil sampling

The study area is located at an abandoned coking plant industrial park (N 39°53′ ~ 39°59′, E 116°07′ ~ 116°14′) in Beijing, China, which has a long land-use history (Figure 1A). According to historical data, the main pollutants in this area are BTEXs and polycyclic aromatic hydrocarbons. Meanwhile, the highly contaminated area, lightly contaminated area and uncontaminated area are divided as Figure 1B. Ten soil samples were randomly collected from the highly contaminated area, and eight soil samples were randomly collected from the lightly contaminated area. For comparison, five control soil samples were also obtained from uncontaminated area. Considering the soil heterogeneous, three parallel samples from each sampling site were mixed as a single sample for analysis (Xu et al., 2022). The sampling depth of all samples is listed in Supplementary Table S1.

Each soil sample was separated into two parts: one part was packed into a sealed bag for the analysis of soil physicochemical parameters, soil lithology and microbial characteristics, and the other was put in a brown glass bottle for the analysis of petroleum hydrocarbon concentrations. Both the sealed bags and the bottles were labeled according to the sampling site and then transported back to the laboratory in a 4°C storage box. After removing the stones and plant debris, the soil samples in sealed bags were divided into two parts. One part was sieved through a 2 mm screen and stored at 4°C for further physicochemical and lithology analyses, and the other part was stored at −80°C for molecular biological analysis. The soil samples in the brown bottles were stored at 4°C for contamination analysis.

### 2.2. Soil physicochemical analysis

Soil lithology was be determined according to Xu et al. (2022). Soil physicochemical parameters, including pH, moisture, total carbon (TC), total nitrogen (TN), nitrate (NO_3_^−^), nitrite (NO_2_^−^) and ammonium (NH_4_^+^), were examined according to previous report (Yuan et al., 2021).

In detail, the soil lithology was determined by Laser particle size analyzer (Mastersizer 2000, Britain), the pH was determined using a pH analyzer with a 1:5 soil/water mixture (HI2221, Italy). The moisture content was determined by oven-drying 2 g fresh soil at 105°C until it reached a constant weight. The TC and TN contents were determined with an elemental analyzer (Elementar, Germany). NH_4_^+^, NO_2_^−^ and NO_3_^−^ which were extracted from a 1:5 fresh soil/2 M KCl mixture, were determined with a flow injection analyzer (AACE, Germany). The soil lithology of all samples is listed in Supplementary Table S1. The physicochemical parameters of all samples are listed in Supplementary Table S2.

The details and classified information of petroleum hydrocarbons detected in this study are listed in Supplementary Table S9. The concentrations of petroleum hydrocarbons were analyzed according to the methods of the Ministry of Ecology and Environmental Protection with total petroleum hydrocarbons, TPH (C6-C9) using HJ 1020–2019, TPH (C10-C40) using HJ 1021–2019, BTEX using HJ 605–2011, and PAHs using HJ 834–2017. All of the characteristics of each soil sample were determined in triplicate. The contaminant compositions and concentrations of all samples are listed in Supplementary Tables S3, S5, S7. The corresponding quality control information in the process of determination are listed in Supplementary Tables S4, S6, S8.

### 2.3. Soil multifunctionality assessment

Soil ecological functions include many aspects (such as nutrient cycling, primary production, etc.). Therefore, soil ecological functions should be evaluated according to various soil functional variables rather than a single variable, which is soil multifunctionality. In this study, soil multifunctionality was calculated according to the averaging approach (Qiu et al., 2021). Soil multifunctionality was assessed by multi-soil functional variables (including ammonia, nitrate, total nitrogen, total carbon, moisture and pH). The data of each soil functional variable were standardized from 0 to 1 by the “Z score” method and “Max-Min” method. Then, the conversion results of various soil functional data were averaged to obtain soil multifunctionality for each soil samples. The calculation formula is as Equations 1.


(1) $$Mi=\frac{1}{N}\overset{N}{\underset{j=1}{\sum }}\left\{\begin{array}{l} \left[\frac{x_{ij}-u_{j}}{\partial_{j}}-{\left(\frac{x_{ij}-u_{j}}{\partial_{j}}\right)}_{\min}\right]/\\ \left[{\left(\frac{x_{ij}-u_{j}}{\partial_{j}}\right)}_{\max}-{\left(\frac{x_{ij}-u_{j}}{\partial_{j}}\right)}_{\min}\right] \end{array}\right\}$$

where Mi is the soil multifunctionality of sample i , N is the number of soil functional variable, $x_{ij}$ is the actual measured value of soil functional variable j of sample i , $u_{j}$ is the mean value of the soil functional variable j in all soil samples, $\partial_{j}$ is the standard deviation of soil functional variable j in all soil samples.

### 2.4. DNA extraction and high-throughput sequencing

The E.Z.N.A.® soil DNA Kit (Omega Bio-tek, Norcross, GA, U.S.) was used to extracted microbial DNA. Extraction procedure strictly followed the kit instructions. After extraction, 1% agarose gel was used to test the quality of DNA extracted. Nextly, region V3-V4 of the bacterial 16S rRNA gene was amplified with primer pairs 338F (5’-ACTCCTACGGGAGGCAGCAG-3′) and 806R (5’-GGACTACHVGGGTWTCTAAT-3′). The amplification condition was as follows: initial denaturation at 95°C for 3 min, followed by 27 cycles of denaturing at 95°C for 30 s, annealing at 55°C for 30 s and extension at 72°C for 45 s, and single extension at 72°C for 10 min, and end at 4°C. After amplification, 2% agarose gel was used to test the quality of amplification products. Lastly, the Illumina MiSeq PE300 platform (Illumina, San Diego, USA) was used to determinate the sequences of amplification product.

The raw sequences were quality filtered by fastp^1^ (version 0.20.0) and merged by FLASH version 1.2.7. Then the sequences obtained were clustered by USEARCH7-uparse. Similarity threshold was set as 0.97. Clustering results were compared with silva138/16s_bacteria database (using confidence threshold of 0.7) to obtain the species taxonomic information. The quality control information of sequences is presented in Supplementary Table S10.

The raw sequencing data obtained in this research were submitted to the NCBI Sequence Read Archive (SRA) under accession number PRJNA890419, which can be found by the “reviewer” link: https://dataview.ncbi.nlm.nih.gov/object/PRJNA890419?reviewer=sbito8l9ubnvsfslt1jistntjb

### 2.5. Bioinformatics analysis

The alpha diversity (Simpson index and Chao index), beta diversity (PCoA analysis) and variance partitioning analysis (VPA) were obtained from the major cloud platform.^2^ The interspecies correlation and *p* value of the microbial community was calculated by Networkx. The co-occurrence network was constructed, analyzed and visualized using Gephi 0.9.3. Network module was divided according to previous literatures (Faust et al., 2012; Williams et al., 2014; Dubin et al., 2016). The heatmaps between TPH and environmental factors were generated with the R package “pheatmap.” The niche breadth of keystone taxa was obtained according to Levins (1968). The niche overlap of keystone taxa was obtained according to Pianka (1973).

### 2.6. Statistical analysis

The data were analyzed in Excel 2016 and R 3.6.1. Significant differences of dominant taxa and keystone taxa in highly contaminated, lightly contaminated and uncontaminated soils were obtained by the Kruskal–Wallis rank sum test. Significant differences in the beta diversity of the microbial community in highly contaminated, lightly contaminated and uncontaminated soils were obtained by Adonis. Significant differences in alpha diversity or network topological parameters in highly contaminated, lightly contaminated and uncontaminated soils were obtained by the Wilcoxon rank sum test. The graphics were drawn by the software Origin2020 and R 3.6.1.

## 3. Results

### 3.1. Soil characteristics and multifunctionalities

Soil provides major functions for Earth’s ecosystem (Xu et al., 2008). Petroleum hydrocarbons at different concentrations can significantly change the soil microbial community and soil physiochemical properties, thus changing the soil ecological function (Li et al., 2020; Chen et al., 2022; Gao et al., 2022). Therefore, to explore the effects of petroleum hydrocarbon concentration on soil multifunctionality, the physiochemical parameters of the soil were determined to calculate soil multifunctionality, and the concentration of petroleum hydrocarbons was determined.

**Figure 2:**
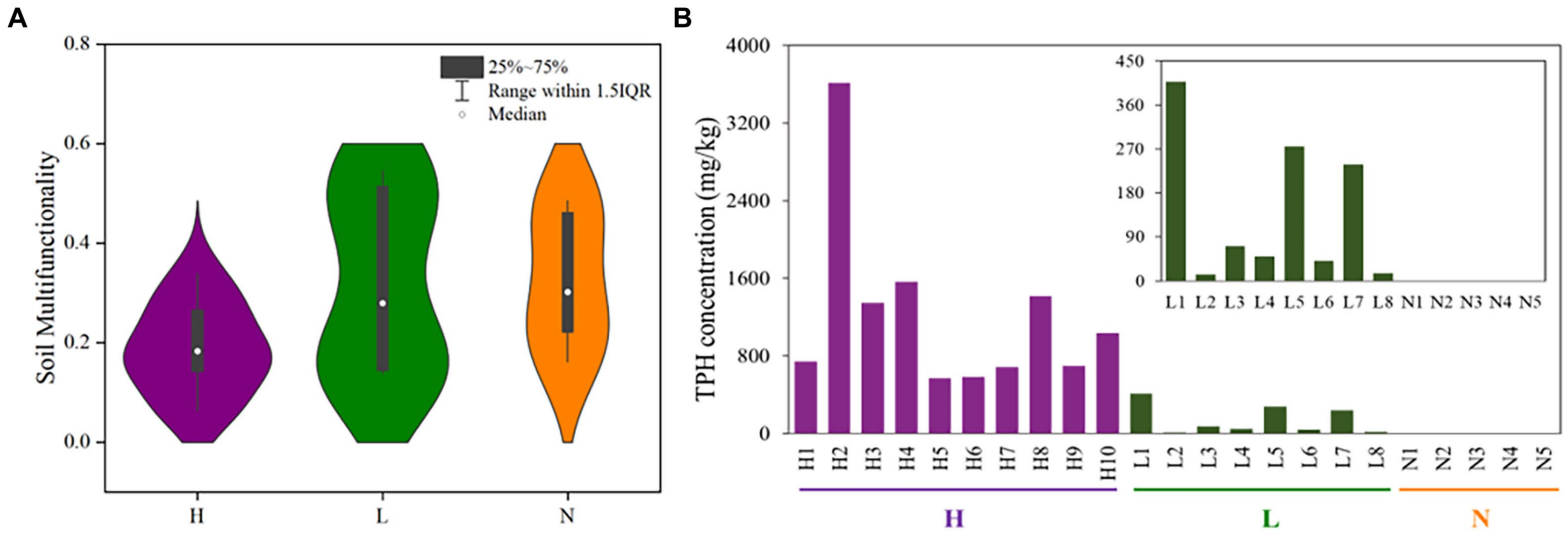
The multifunctionalities and total petroleum hydrocarbon concentrations in highly contaminated soils (H), lightly contaminated soils (L) and uncontaminated soils (N). **(A)** Distribution characteristics of soil multifunctionality based on Violin with box (after getting rid of an outlier in highly contaminated soils). **(B)** Total petroleum hydrocarbon concentrations of soil samples.

The results showed that the petroleum hydrocarbon concentrations of ten highly contaminated soil samples were 565–3613 mg•kg^−1^, and the petroleum hydrocarbon concentrations of eight soil samples in the lightly contaminated area were 13–408 mg•kg^−1^ (Figure 2B). The multiple ecological functionalities of highly contaminated soils were strongly clustered, which explained 64.7 percent of the variation. In addition, the soil multifunctionalities were significantly decreased in highly petroleum hydrocarbon-contaminated soils, while the multifunctionalities of some samples in lightly contaminated soils were improved (Figure 2A; Supplementary Figure S1a).

Among all soil samples, TPH was significantly negatively correlated with nitrite and TC (*p* < 0.05). In lightly contaminated soils, TPH was significantly positively correlated with TN and moisture (*p* < 0.01; Supplementary Figure S1b).

### 3.2. Dominate microbes of soil microbial communities

The soil microbial community are sensitively regulated by soil physiochemical properties and petroleum hydrocarbons at different concentrations (Sheng et al., 2021; Gao et al., 2022; Xu et al., 2022). Thus, the microbial community composition in all soil samples was explored by 16S high-throughput sequencing. In addition, the influence factors for the differences in microbial composition were analyzed by VPA.

**Figure 3:**
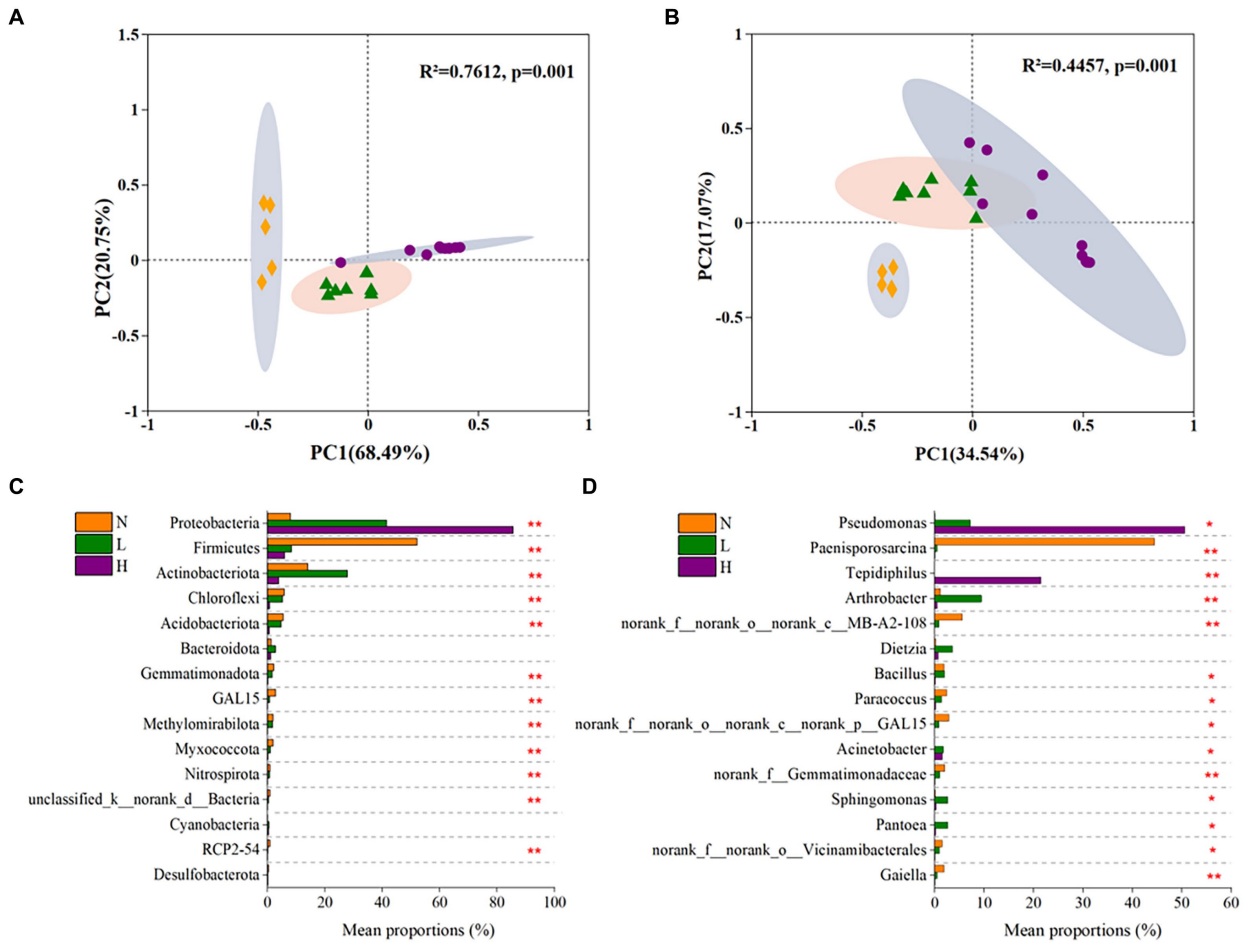
The beta diversity of the microbial community and significant differences of in dominant microbes in highly contaminated soils (H), lightly contaminated soils (L) and uncontaminated soils (N). The beta diversity of the microbial community at the phylum level **(A)** and genus level **(B)** was explored by principal coordinate analysis (PCoA) based on Bray–Curtis dissimilarity and Adonis analysis. Significant differences in phyla **(C)** and genus **(D)** whose relative abundance belonged to the top 15 in the microbial community. The Kruskal–Wallis rank-sum test was used to calculate significant differences. *p* was obtained after fdr adjustment and Tukey–Kramer *post hoc* test (*p < 0.05; **p < 0.01; ***p < 0.001).

The results showed that all of the sequencing data obtained from 23 soil samples were assigned to 56 phyla, 168 classes, 405 orders, 667 families and 1,368 genus. The microbial communities were strongly clustered (*p* = 0.001) according to the petroleum hydrocarbon contamination levels, which explained 89.24 and 51.61% of the total variance at the phylum level and genus level, respectively (Figures 3A,B).

At the phylum level, the microbial communities in all soil samples were mainly composed of Proteobacteria (ranging from 4.88 to 98.96% in each soil sample), Firmicutes (0.95–75.47%), Actinobacteria (0–40.47%), Chloroflexi (0.16–9.59%), Acidobacteria (0–13.51%) and so on (Supplementary Figure S2a). As the concentration of petroleum hydrocarbons increased, the relative abundance of Proteobacteria significantly increased, while the relative abundances of Firmicutes, Chloroflexi and Acidobacteria significantly decreased. In addition, the relative abundance of Actinobacteria in lightly contaminated soils was higher than that in other soils (Figure 3C).

At the genus level, the microbial communities mainly comprised *Pseudomonas* or *Tepidiphilus* (the sum of the two ranging from 14 to 95.8%) in highly contaminated soils, *Paenisporosarcina* (ranging from 15.3 to 67.3%) in uncontaminated soils. While the relative abundance of most genus in lightly contaminated soils was uniform (Supplementary Figure S2b). The relative abundance of *Paenisporosarcina* (*p* < 0.01)*, norank_f__norank_o__norank_c__MB-A2-108* (*p* < 0.01) and *Paracoccus* (*p* < 0.05) was highest in uncontaminated soils. While the relative abundance of *Arthrobacter* (*p* < 0.01), *Sphingomonas* (*p* < 0.05) and *Pantoea* (*p* < 0.05) was highest in lightly contaminated soils. The relative abundance of *Pseudomonas* (*p* < 0.05) and *Tepidiphilus* (*p* < 0.01) was highest in highly contaminated soils (Figure 3D).

The results of VPA showed petroleum hydrocarbons, soil physicochemical properties and soil lithology accounted for 69.04% of the total microbial community variation. Therein, petroleum hydrocarbons alone accounted for 45.80% of the microbial community variation, far higher than that of soil physicochemical properties (26.37%) and soil lithology (0%) alone (Supplementary Figure S3). Among various explanatory factors, high molecular weight polycyclic aromatic hydrocarbons were the most important in contaminants, while ammonia was the most important in soil physicochemical parameters (Supplementary Table S11).

### 3.3. Microbial diversity and its relationship with soil multifunctionality

Soil microbial diversity is crucial to maintaining the multifunctionality of soil ecosystems (Delgado-Baquerizo et al., 2016; Qiu et al., 2021). The loss of biodiversity will lead to the impairment of natural ecosystem functions and the diminishment of the number and quality of services they provide (Cardinale et al., 2012). According to previous reports, microbial community diversity in soil can be increased (Christopher and Christopher, 2004) or reduced (Vinas et al., 2005; Bordenave et al., 2007) by petroleum hydrocarbon contamination. To explore the effects of petroleum hydrocarbons at different concentrations on microbial diversity, the alpha diversity of each soil sample was calculated.

**Figure 4:**
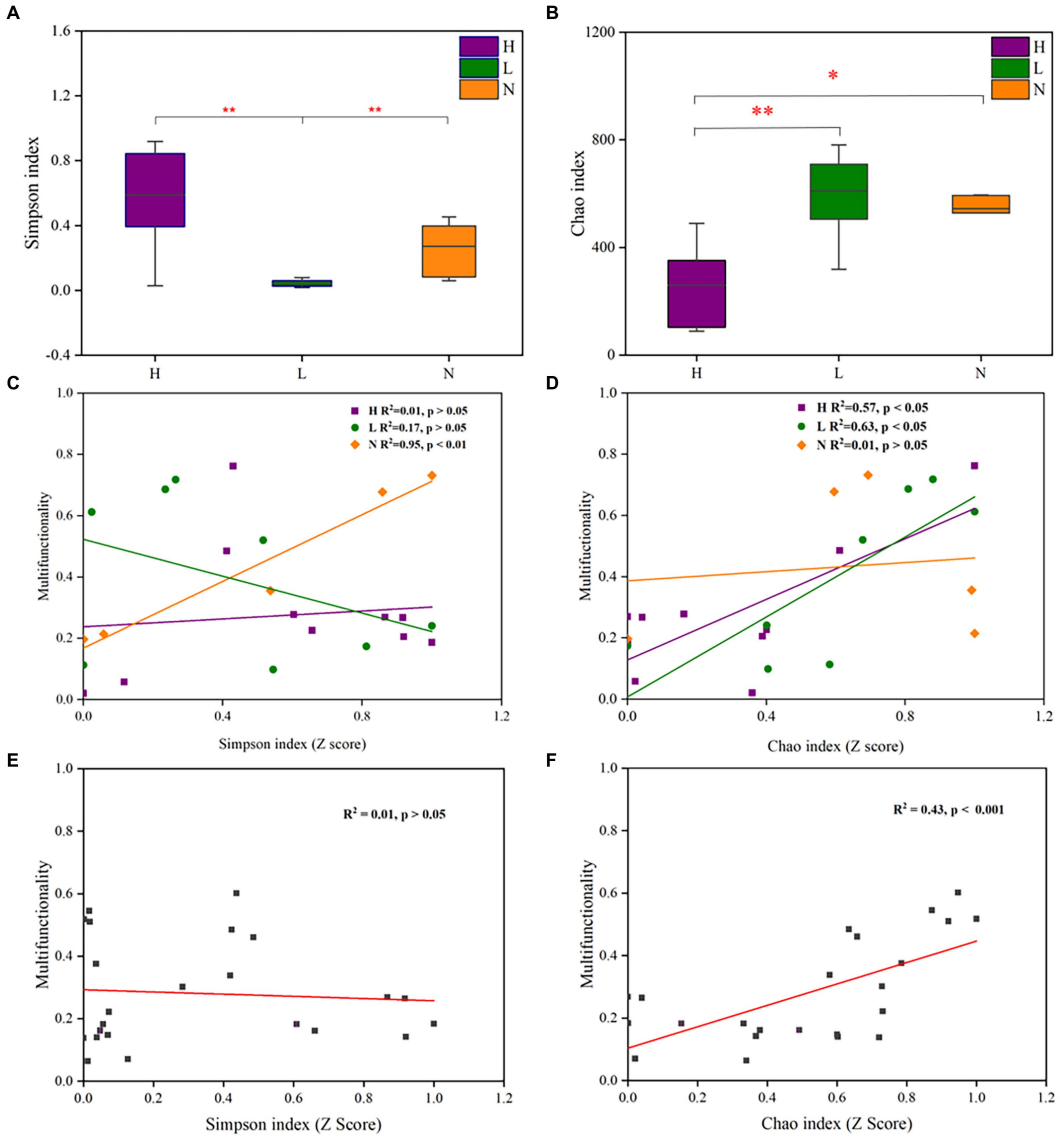
Alpha diversity of the microbial community and its relationship with soil multifunctionality in soils with different petroleum hydrocarbon contamination levels. The Simpson index **(A)** and Chao index **(B)** of the microbial community in highly contaminated soils (H), lightly contaminated soils (L) and uncontaminated soils (N). The Pearson correlation between the Simpson index **(C)** or Chao index **(D)** and soil multifunctionality in highly contaminated soils (H), lightly contaminated soils (L) and uncontaminated soils (N). The Pearson correlation between the Simpson index **(E)** or Chao index **(F)** and soil multifunctionality in all soil samples. The Wilcoxon rank-sum test was used to calculate significant differences (*p < 0.05; **p < 0.01; ***p < 0.001).

The Simpson index reflects both the richness and evenness of the microbial community, and the Chao index reflects microbial community richness. The results showed that the Simpson index of the microbial community in lightly contaminated soils was significantly lower than that in the uncontaminated soils (*p* < 0.01). Meanwhile, the Chao index of the microbial community in highly contaminated soils was significantly lower than that in the uncontaminated soils (*p* < 0.05; Figures 4A,B). The Simpson index was positively (*p* < 0.01) correlated with soil multifunctionality in uncontaminated soil (Figure 4C). The Chao index was significantly positively correlated (*p* < 0.05) with soil multifunctionality in both highly and lightly contaminated soils (Figure 4D). In addition, there was a significant positive correlation (*p* < 0.001) between the soil multifunctionality and the Chao index of the microbial community (Figures 4E,F). Therefore, it can be concluded that soil multifunctionality was enhanced in lightly contaminated soils and was reduced in highly contaminated soils.

### 3.4. Microbial network complexity and its relationship with soil multifunctionality

The interactions among microbes are crucial to maintain the structure and stability of the soil microbial community (Hallam and McCutcheon, 2015; Shi et al., 2016). These interactions can be explored by co-occurrence network analysis, which has been demonstrated to be a powerful tool that can identify important ecological units and keystone taxa in microbial communities (Barberán et al., 2012; Geng et al., 2020, 2022; Xu et al., 2022).

**Figure 5:**
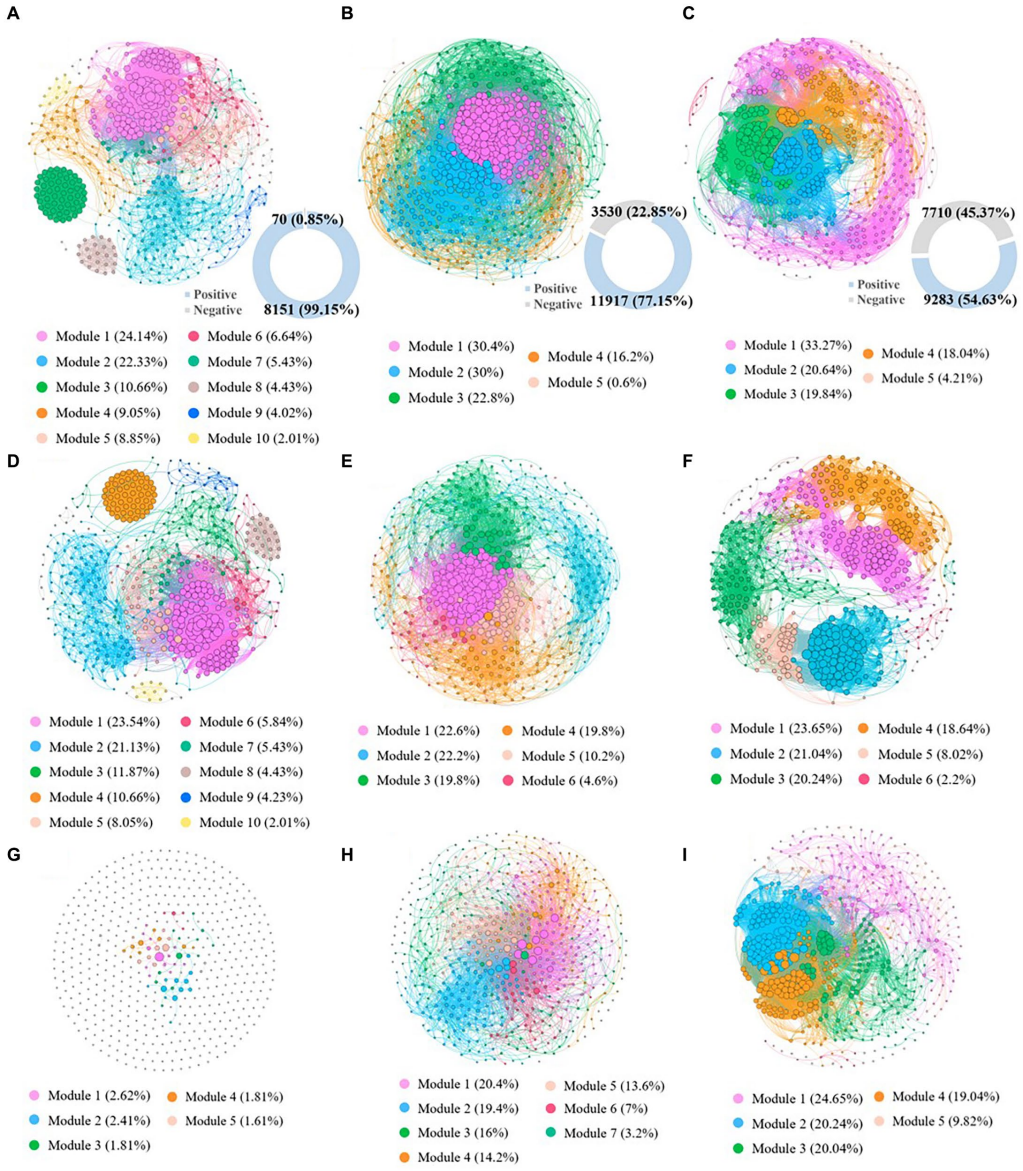
The co-occurrence patterns of the microbial community based on genus in highly contaminated soils **(A)**, lightly contaminated soils **(B)** and uncontaminated soils **(C)**. Positive association co-occurrence network of the microbial community based on genus in highly contaminated soils **(D)**, lightly contaminated soils **(E)** and uncontaminated soils **(F)**. Negative association co-occurrence network of the microbial community based on genus in highly contaminated soils **(G)**, lightly contaminated soils **(H)** and uncontaminated soils **(I)**. Only nodes that were significantly correlated (Spearman’s > 0.75; *p* < 0.05) with each other were connected by edges. The sizes of the nodes are proportional to the number of connections. The nodes were colored based on microbial modularity class. Figures in blue rings represent the number of positive edges, and figures in grey rings represent negative edges.

In this study, co-occurrence networks were constructed at the genus level in highly contaminated, lightly contaminated and uncontaminated soils to investigate the co-occurrence patterns of the microbial community. The network topology parameters are described in Supplementary Table S12. The number of network links in highly contaminated soils (8221) was significantly lower than that in lightly contaminated soils (15447) and uncontaminated soils (16993). In addition, with the increase in petroleum hydrocarbon concentration, the proportion of negative links in the network decreased significantly. The number of network negative links in highly contaminated soils (70) was lower than that in lightly contaminated soils (3530) and uncontaminated soils (7710). While the number of network positive links in lightly contaminated soils (11917) was higher than that in highly contaminated soils (8151) and uncontaminated soils (9284), which was consistent with the change rule of microbial diversity among highly contaminated soils, lightly contaminated soils and uncontaminated soils. Furthermore, the results of topology parameters of positive association co-occurrence network indicated, the network modularity coefficients were 0.53, 0.31 and 0.62 in highly contaminated, lightly contaminated and uncontaminated soils, respectively. Network hubs are defined as nodes with a high degree in the network (bacteria >50; Geng et al., 2022). There were 163 hubs in highly contaminated soils, 177 hubs in lightly contaminated soils and 150 hubs in uncontaminated soils. Positive association co-occurrence network can reflect the synchronous response of microbes to environmental changes and can better reflect the interspecific interactions (de Vries et al., 2018). Therefore, the results indicated that the network connectivity and complexity were significantly reduced in highly petroleum hydrocarbon-contaminated soils, while were increased in lightly petroleum hydrocarbon-contaminated soils. In addition, high petroleum hydrocarbon contamination reduced the concentrated interactions of soil microbes, while light petroleum hydrocarbon contamination might increase the concentrated interactions of soil microbes (Figure 5).

The degree is the number of all connected edges of a node, reflecting the associations among microbes (Proulx et al., 2005; Banerjee et al., 2018). Closeness centrality refers to the distance of one node to all other nodes and reflects the importance of a node for information dissemination (Ma'Ayan, 2011). Therefore, the degree and closeness centrality based on the node level were used to assess the connectivity and clustering of the microbial co-occurrence network to explore the correlation between the soil microbial co-occurrence network and soil multifunctionality.

**Figure 6:**
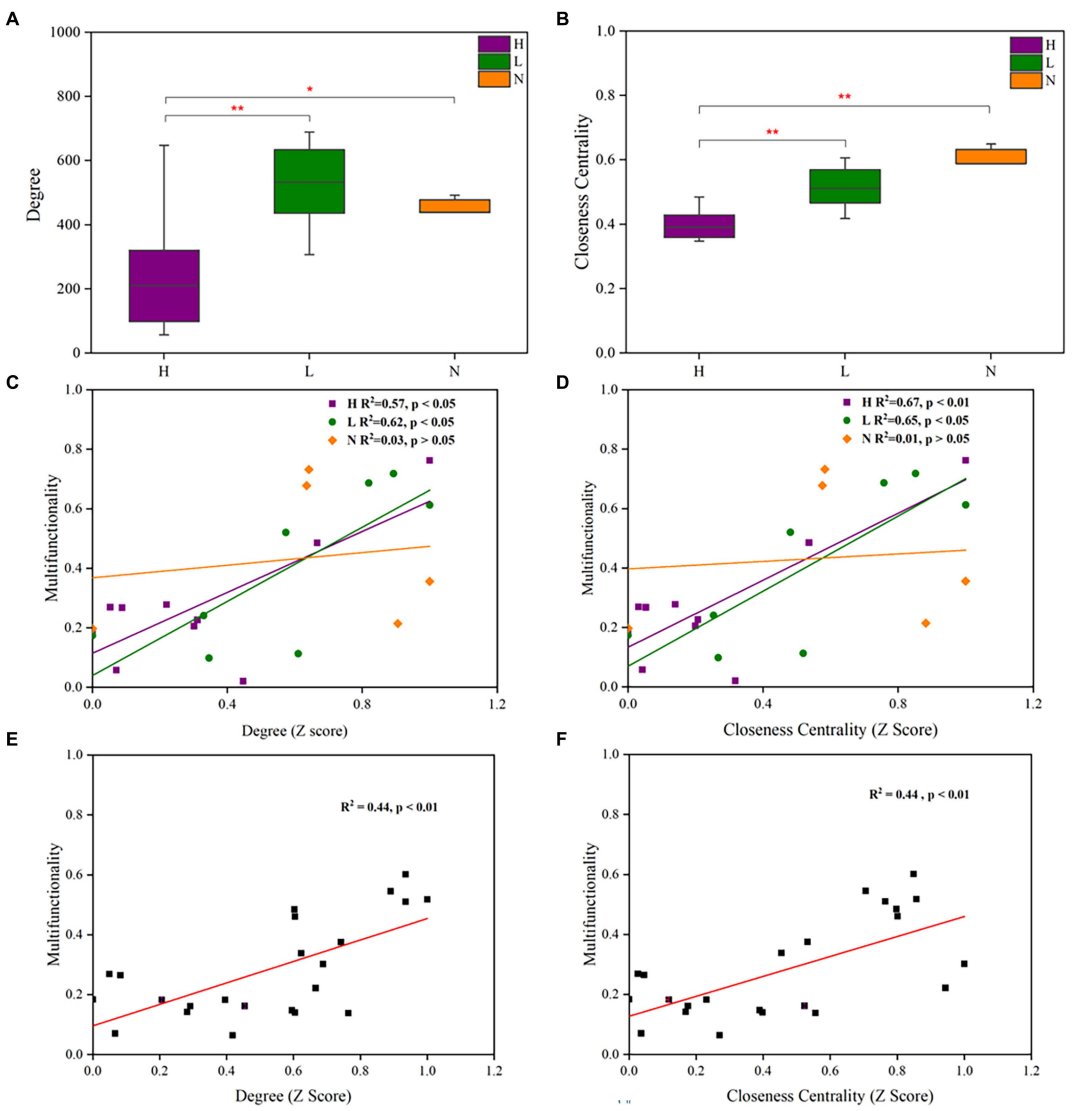
Topological parameters of microbial co-occurrence networks and their relationship with soil multifunctionality in soils with different petroleum hydrocarbon contamination levels. The degree **(A)** and closeness centrality **(B)** of microbial co-occurrence networks in highly contaminated soils (H), lightly contaminated soils (L) and uncontaminated soils (N). The Pearson correlation between degree **(C)** or closeness centrality **(D)** and soil multifunctionality in highly contaminated soils (H), lightly contaminated soils (L) and uncontaminated soils (N). The Pearson correlation between degree **(E)** or closeness centrality **(F)** and soil multifunctionality in all soil samples. The Wilcoxon rank-sum test was used to calculate significant differences (*p < 0.05; **p < 0.01; ***p < 0.001).

The results showed that high concentrations of petroleum hydrocarbons significantly reduced the degree (*p* < 0.05) and closeness centrality (*p* < 0.01) of the soil microbial networks (Figures 6A,B). In addition, in highly or lightly contaminated soils, the degree and closeness centrality were positively correlated with soil multifunctionality (*p* < 0.05). In uncontaminated soils, there was no correlation between microbial network parameters and soil multifunctionality (*p* > 0.05) (Figures 6C,D). The degree and closeness centrality were positively correlated (*p* < 0.01) with soil multifunctionality (Figures 6E,F), indicating that petroleum hydrocarbon contamination decreased soil multifunctionality.

### 3.5. Keystone taxa of the microbial network and its niche characteristics

Keystone taxa that have the characteristics of high degree, low betweenness centrality, and high closeness centrality play a critical role in microbial community structure and function and serve as indicators of environmental change (Berry and Widder, 2014; Banerjee et al., 2018). Their niche breadth and overlap play a decisive role in species diversity and community structure stability (Venjakob et al., 2016). Therefore, we identified the keystone taxa of the microbial community and explored their niche characteristics.

**Figure 7:**
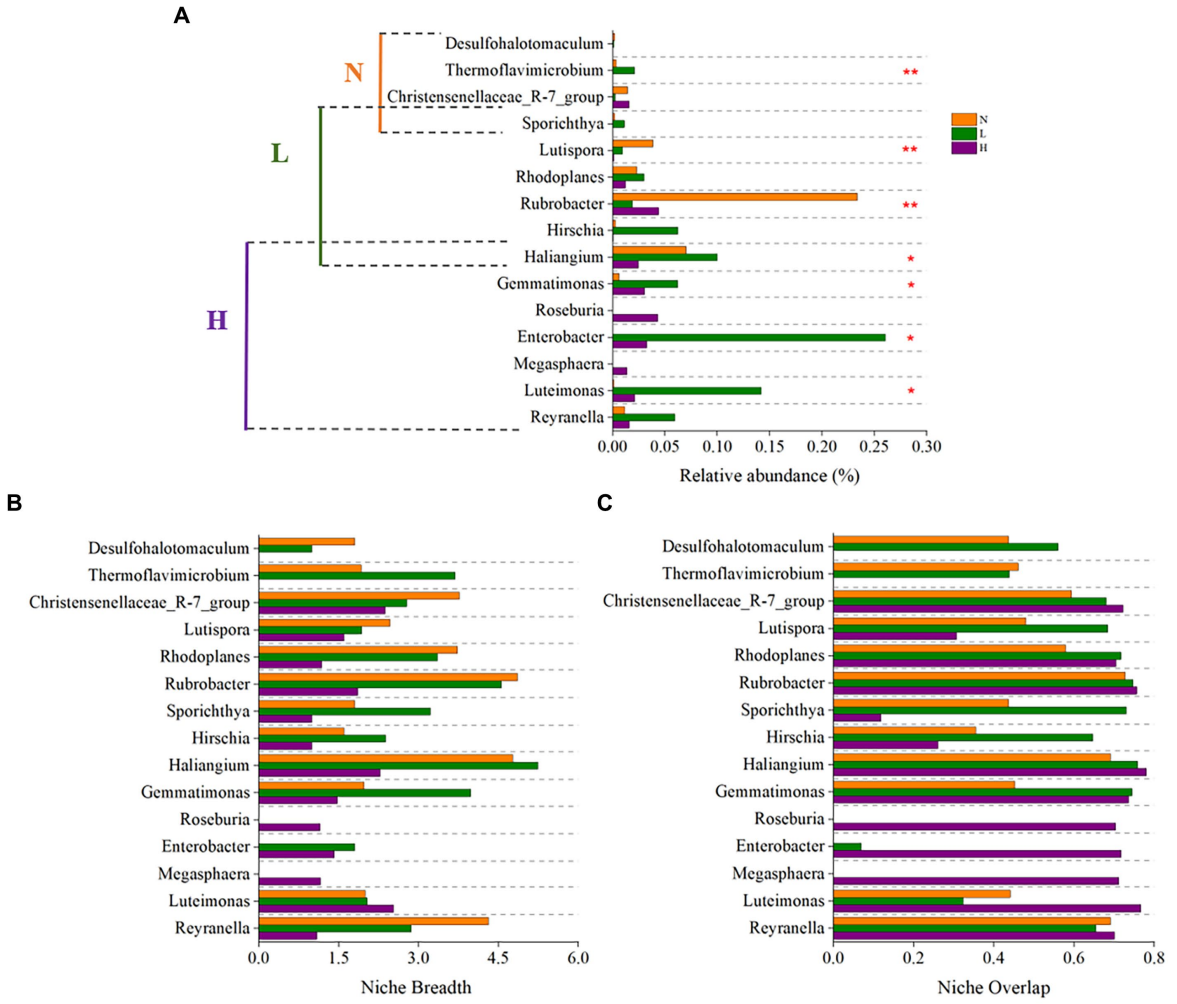
The relative abundance **(A)**, niche breadth **(B)**, and niche overlap **(C)** of keystone taxa in highly contaminated soils (H), lightly contaminated soils (L) and uncontaminated soils (N).

The results showed that the keystone genus in the highly contaminated soils were *Reyranella, Luteimonas, Megasphaera, Enterobacter, Roseburia, Gemmatimonas, Haliangium*. The keystone genus in the lightly contaminated soils were *Haliangium, Hirschia, Rubrobacter, Rhodoplanes, Lutispora, Sporichthya*. The keystone genus in the uncontaminated soils were *Sporichthya, Christensenellaceae_R-7_group, Thermoflavimicrobium, Desulfohalotomaculum* (Figure 7A). All of the above 15 keystone genus were not rich species in the community (relative abundance <1%), this is consistent with the previous report (Pester et al., 2010; Liang et al., 2016; Shi et al., 2016; Yang et al., 2018; Ma et al., 2021). Because the taxa with low relative abundance usually adopt K strategy and have closer ecological associations when faced with environmental stress (Pester et al., 2010; Shu et al., 2018). Compared to other taxa, the keystone taxa often play an important role in maintaining network structure out of proportion of their relative abundance (Shi et al., 2016).

Most of the keystone taxa of lightly contaminated soils and uncontaminated soils had widest niche breadth in uncontaminated soils, while most of the keystone taxa of highly contaminated soils had widest niche breadth in highly contaminated soils or lightly contaminated soils. In terms of niche overlap, the niche overlap of almost all keystone genus in highly contaminated soils were highest (Figures 7B,C).

## 4. Discussion

Petroleum hydrocarbon contamination in soil is an important and universal environmental change caused by human activities that has an important impact on soil ecological functions. Our research focused on the impact of petroleum hydrocarbon contamination on soil microbial ecology and the corresponding soil ecological functions. By determining the composition, diversity, co-occurrence patterns and keystone taxa of the microbial community at different contamination levels in soils, this study demonstrated that contamination shifts the composition of the microbial community in soil. The microbial diversity is increased in lightly contaminated soils but reduced in highly contaminated soils. High contamination simplifies microbial networks while light contamination may enhance microbial interactions. Furthermore, contamination may enhance microbial interspecific cooperation, altering the niche characteristics of keystone taxa in microbial networks. In addition, microbial characteristics in petroleum hydrocarbon contaminated soil are related to soil multifunctionality. This study found that the response of the microbial community to petroleum hydrocarbon stress may show a threshold effect, which is of great significance for gaining insight into the biological mechanisms of the actual contamination site.

### 4.1. Succession of microbial community composition occurs from lightly petroleum hydrocarbon-contaminated soils to highly contaminated soils

In soils contaminated by petroleum hydrocarbons for a long time, light organic molecules with high bioavailability have been almost exhausted, and the organic matter in the soil is mostly resistant to biodegradation (Sheng et al., 2021). Therefore, the relative abundance of microbes that could directly utilize a large amount of refractory organic matter in soil has significantly increased, such as *Pseudomonas*, *Tepidiphilus* and *Sphingomonas*, which are responsible for degrading aliphatic and aromatic compounds (Huang et al., 2019, 2023; Ling et al., 2023). High concentration petroleum hydrocarbons cause the oxygen-deficient environment in soil. Therefore, the relative abundance of microbes with denitrification potential increased, such as *Arthrobacter* and *Paracoccus* (Zhang et al., 2018; Zheng et al., 2018). The relative abundance of some microbes which are related to degradation of organic matter was significantly decreased, such as Firmicutes, which was reported to be mainly responsible for degrading plant polymers (Brzeszcz and Kaszycki, 2018). While in contaminated soils, it is difficult for plants to survive (Xu et al., 2022).

Although both the dominant genus of microbial communities in light and high contamination soils had potential functions related with petroleum hydrocarbon degradation and denitrification, the composition and relative abundance of dominant genus were completely different. The possible reason is that low concentrations of petroleum hydrocarbons provide available carbon sources for certain soil microbes. Conversely, high concentrations of petroleum hydrocarbons stress pressure on these microbes (Lindgren et al., 2014; Xu et al., 2018). Further reasons need to be explored by analyzing the diversity and co-occurrence patterns of microbial communities.

### 4.2. Microbial diversity is increased in lightly petroleum hydrocarbon-contaminated soils but reduced in highly contaminated soils

Soil microbial community diversity and soil multifunctionality decreased in highly contaminated soils, which was similar to the results of Gao et al. (2022). This is because petroleum hydrocarbons at high concentrations can poison the soil microbial ecosystem and inhibit the growth and metabolism of microbes. Only the growth of certain microbes that can adapt to environments contaminated by petroleum hydrocarbons at high concentrations can be stimulated (Xu et al., 2022). In addition, the mineralization of petroleum hydrocarbons limits the utilization of electron acceptors by microbes. High concentrations of petroleum hydrocarbons also increase the ratio of carbon to nitrogen in soil, causing intense competition among microbes for nitrogen sources, leading to the rapid growth of certain microbes that have the ability to fix nitrogen and a decrease in microbial community diversity (Zhou and Crawford, 1995; Bordenave et al., 2007; Ma et al., 2021).

Inversely, low concentrations of petroleum hydrocarbons could increase microbial diversity, consistent with the ecological theories-intermediate disturbance hypothesis (IDH), which states that when an ecosystem is moderately disturbed, its species diversity will be the highest (Gerwing et al., 2017; Wang et al., 2018; Liu et al., 2019; Swart et al., 2019). This is because low concentrations of petroleum hydrocarbons provide available carbon sources and energy for microbes in soil and stimulate the growth of microbes that can degrade petroleum hydrocarbons (Xu et al., 2018). In lightly contaminated soils, more high molecular weight petroleum hydrocarbons may promote the production of more secondary products, thus promoting microbial diversity.

### 4.3. High petroleum hydrocarbon contamination simplifies microbial networks, while light contamination enhances microbial networks

Our results showed that long-term high petroleum hydrocarbon contamination led to a decrease in links in microbial positive association co-occurrence networks. While light petroleum hydrocarbon contamination led to an increase in links in microbial positive association co-occurrence networks. This is consistent with previous reports (Sheng et al., 2021; Gao et al., 2022). High petroleum hydrocarbon contamination leads to differentiation of the microbial niche, simplification of the species structure, and a reduction in interspecific competition (Ma et al., 2021). A possible reason is that high concentrations of petroleum hydrocarbons in soils greatly limit the available resources for microbes (Qiu et al., 2021). In contrast, the microbial interactions may be enhanced in lightly contaminated soils with diversified carbon resources. This phenomenon conformed to the basic ecological principle of the stress-gradient hypothesis (SGH) (Bertness and Callaway, 1994).

In addition, the links in co-occurrence networks are related to both interactions among species and environmental filtering (Hernandez et al., 2021). Therefore, with the increase of petroleum hydrocarbon concentration, the proportion of positive links in microbial network increases for two reasons. One is petroleum hydrocarbon stress results in the replacement of “competitive” species by symbiotic species in the community (Durán et al., 2018). Because a neighbor-buffering strategy was adopted by soil microbial community to jointly resist the stress effect of petroleum hydrocarbons. The other is petroleum hydrocarbon contamination promotes the similarity of ecological functions among microbes (Chaffron et al., 2010). Because most dominant species in the community had the function of degrading petroleum hydrocarbons.

It has been reported that the weakening of negative interactions in microbial networks is detrimental to community stability (Coyte et al., 2015; Hernandez et al., 2021). While the proportion of negative interactions in microbial network may decrease with the increase of petroleum hydrocarbon concentration. This suggests that petroleum hydrocarbon stress may lead to a reduction in the stability and complexity of soil microbial communities. This conclusion was shared by the vast majority of previously published articles (Liang et al., 2016; de Vries et al., 2018; Huang et al., 2021; Gao et al., 2022). This result was also consistent with the results reflected by other network topology parameters of our co-occurrence network. In addition, high concentration petroleum hydrocarbon stress enhanced modularity coefficient of microbial networks, suggesting that high concentration petroleum hydrocarbons induce a more deterministic approach to microbial community construction (Geng et al., 2020; Sheng et al., 2021; Geng et al., 2022).

### 4.4. Low petroleum hydrocarbon contamination widens the niche breadth of keystone taxa, while high contamination promotes the niche overlap of keystone taxa

All keystone taxa found in our study had strong correlations and mutualistic symbiosis with other species in the community, mainly including three aspects: (1) Metabolites of keystone taxa provide nutrition for other species. It was reported that *Luteimonas, Lutispora, Roseburia, Megasphaera, Reyranella* and *Rhodoplanes* can convert refractory petroleum hydrocarbons into simple molecules (Zhou et al., 2015; Li J. et al., 2018; Pichler et al., 2020; Damtie et al., 2021; Zhang et al., 2022; Abudureheman et al., 2023). (2) Keystone taxa improve the living environment of other species. *Sporichthya* is a major source of bioorganic carbon for other microbes (Zhang et al., 2021; He et al., 2022); *Thermoflavimicrobium* can produce various microbial metabolic enzyme (Darwesh et al., 2020). (3) Keystone taxa promote the biogeochemical cycle of the soil environment. *Desulfohalotomaculum* can utilize sulfate under anaerobic conditions (Bell et al., 2019). *Enterobacter, Gemmatimonas, Haliangium* and *Rubrobacter* are typical denitrification bacteria (Huang et al., 2015; Li L. et al., 2018; Guan et al., 2022; Hou et al., 2023). The potential ecological function of keystone taxa in uncontaminated soils was carbohydrate degradation. The keystone taxa in highly contaminated soils and lightly contaminated soils were mainly composed of some microbes that have the potential to degrade refractory organic matter and utilize nitrate under anaerobic condition.

Petroleum hydrocarbon contamination significantly affected the niche breadth and niche overlap of keystone taxa in the community, which is consistent with the report of Ribeiro et al. (2013), indicating that the key ecological mechanisms of the community were significantly affected by petroleum hydrocarbon contamination. In our study, keystone taxa in the community had a wide niche breadth in uncontaminated and lightly contaminated soils, which was due to their strong adaptability and high resource utilization ability in specific soil environments. With the increase in petroleum hydrocarbon concentration, the niche breadth of the original keystone taxa in the soil gradually narrowed because they could not adapt to the toxic effect caused by high concentrations of petroleum hydrocarbons, while the niche breadth of some microbes that can withstand harsh conditions gradually widened because they had better adaptability to high concentrations of petroleum hydrocarbons. This explains the succession of soil microbial communities with the increase in petroleum hydrocarbon concentration. High concentrations of petroleum hydrocarbons increased niche overlap among keystone genus. This is because with the increase in petroleum hydrocarbon concentration, the resources became concentrated, new keystone microbes in the community had similar resource needs, and the competition among species intensified.

## 5. Conclusion

According to the comparisons of the relationships between the ecological function and microbial characteristics of contaminated soils and uncontaminated soils of aged realistic contaminated fields, we demonstrate that high petroleum hydrocarbon contamination indeed results in a decline in soil ecological function, while light contamination might result in an increase in soil ecological function. 16S high-throughput sequencing technology combined with biological information analysis of microbes in soils with different contamination levels reveals that petroleum hydrocarbon contamination does not always reduce the relative abundance of microbes and the microbial diversity. Light petroleum hydrocarbon contamination promoted microbial diversity and may enhance microbial interactions. When the concentration of petroleum hydrocarbons increased, the co-occurrence network among microbial communities was simplified, and interspecific cooperation may be strengthened, which meant that the community stability decreased. However, the niche overlap of keystone species increased in highly contaminated soils. In general, we cautiously infer that maybe there is a threshold effect of petroleum hydrocarbons on soil microbes, which can be an important reference for the refined management of petroleum hydrocarbon-contaminated sites. However, due to the high heterogeneity of soil structure and the limitation of co-occurrence network, the results of microbial community interactions obtained from our analysis may be inaccurate, which we plan to further explore in the next step.

## Data availability statement

The data presented in the study are deposited in the NCBI repository, accession number is PRJNA890419, which can be found at this link: https://dataview.ncbi.nlm.nih.gov/object/PRJNA890419?reviewer=sbito8l9ubnvsfslt1jistntjb.

## Author contributions

WJ: investigation, formal analysis, visualization, and writing – original draft. LC: methodology and formal analysis. QT: resources and investigation. YL: investigation and data curation. JD and KY: methodology. QY: validation. SW, JL, and GN: provide sampling site and assist in designing sampling plan. LZ: supervision, methodology, and writing – review and editing. AD: conceptualization, project administration, and funding acquisition. All authors contributed to the article and approved the submitted version.

## Funding

This research was supported by Beijing Municipal Natural Science Foundation (8232037), National Key R&D Program of China (No. 2018YFC1800905), Key Science and Technology Projects of Inner Mongolia autonomous region (2019ZD001), and China Postdoctoral Science Foundation (2021M690428).

## Conflict of interest

SW, JL, and GN are employed by Beijing No.4 Municipal Construction Engineer Co., Ltd.

The remaining authors declare that the research was conducted in the absence of any commercial or financial relationships that could be construed as a potential conflict of interest.

## Publisher’s note

All claims expressed in this article are solely those of the authors and do not necessarily represent those of their affiliated organizations, or those of the publisher, the editors and the reviewers. Any product that may be evaluated in this article, or claim that may be made by its manufacturer, is not guaranteed or endorsed by the publisher.

## Supplementary material

The Supplementary material for this article can be found online at: https://www.frontiersin.org/articles/10.3389/fmicb.2023.1188229/full#supplementary-material

## Footnotes

^1^ https://github.com/OpenGene/fastp

^2^ https://cloud.majorbio.com/
